# Expanded directly binds conserved regions of Fat to restrain growth via the Hippo pathway

**DOI:** 10.1083/jcb.202204059

**Published:** 2023-04-18

**Authors:** Alexander D Fulford, Leonie Enderle, Jannette Rusch, Didier Hodzic, Maxine V Holder, Alex Earl, Robin Hyunseo Oh, Nicolas Tapon, Helen McNeill

**Affiliations:** 1 Department of Developmental Biology, Washington University School of Medicine, St. Louis, United States; 2 Department of Molecular Genetics, University of Toronto, Toronto, Canada; 3 Apoptosis and Proliferation Control Laboratory, The Francis Crick Institute, London, United Kingdom

## Abstract

The Hippo pathway is a conserved and critical regulator of tissue growth. The FERM protein Expanded is a key signalling hub that promotes activation of the pathway, stimulating inhibition of the transcriptional co-activator Yorkie. Previous work defined the transmembrane polarity determinant Crumbs as the primary regulator of Expanded. Here we show that the giant atypical cadherin Fat is essential in regulating Expanded function independently of Crumbs. We show that direct interaction between a highly conserved region of the Fat intracellular domain and Expanded recruits Expanded to the plasma membrane. *In vivo* deletion of Expanded binding regions in Fat causes loss of apical Expanded and promotes tissue overgrowth. In addition, we find that Dachsous binds Fat via interactions of their cytoplasmic domains, and that Expanded can be stabilised by Fat independently of Dachsous binding. These data provide new mechanistic insights into how Fat regulates Expanded, and how Hippo signalling is regulated during organ growth.

## Introduction

The precise and coordinated control of metazoan growth is essential for correctly sized and proportioned adult organisms, and the highly conserved Hippo pathway is key to its regulation. The Hippo pathway modulates growth by inhibiting the transcriptional co-activator Yorkie (Yki). Yki activity is controlled by the core Hippo kinase cassette consisting of the kinases Hippo (Hpo) (Harvey et al., 2003; Jia et al., 2003; Pantalacci et al., 2003; Udan et al., 2003; Wu et al., 2003) and Warts (Wts) (Justice et al., 1995; Xu et al., 1995), which phosphorylates Yki and inhibits its nuclear localisation (Huang et al., 2005). When free from inhibition by these kinases, Yki concentrates in the nucleus where it interacts with transcription factors such as Scalloped (Sd) to promote transcription of cell cycle and anti-apoptotic genes, for example, *cyclinE* and *diap1*, ultimately driving tissue growth (Wu et al., 2008; Zhang et al., 2008). Once Yki is activated, excessive growth is prevented through a negative feedback loop, whereby Yki drives the transcription of its own inhibitors, such as *expanded* (*ex*) (Fulford et al., 2018; Zheng and Pan, 2019).

Upstream of the kinase cassette are numerous inputs into the pathway, such as cell polarity, adherens junctions and the cytoskeleton (Fulford et al., 2018; Zheng and Pan, 2019). One important nexus of signalling is through the 4.1, Ezrin, Radixin and Moesin (FERM) protein Ex. Ex forms a complex with Merlin (Mer) and Kibra (Kib) at the apical junctions (termed the KEM complex), where Ex activates the Hippo kinase cassette by scaffolding core pathway members and by recruiting the scaffold protein Schip1, which promotes Hpo phosphorylation by Tao-1 (Baumgartner et al., 2010; Boggiano et al., 2011; Chung et al., 2016; Genevet et al., 2010; Genevet & Tapon, 2011; Hamaratoglu et al., 2006; McCartney et al., 2000; Poon et al., 2011; Sun et al., 2015; Yu et al., 2010). Ex also directly interacts with WW-domains of Yki at the apical junctions through three PPxY motifs in its C-terminus (Badouel et al., 2009; Oh et al., 2009). This limits the translocation of Yki to the nucleus, as well as bringing it into proximity of the kinase cassette, a process inhibited by Activated cdc42 kinase (Ack) phosphorylation of Ex (Hu et al., 2016). Together these mechanisms promote robust inhibition of Yki function.

Ex is thought to sit at the interface between the major epithelial polarity axes - apico-basal and planar cell polarity (PCP) since it is regulated by two transmembrane proteins, Fat (Ft) and Crumbs (Crb), which organise tissues by regulating both polarity and growth (Fulford et al., 2018; Genevet and Tapon, 2011). The apico-basal polarity protein Crb promotes Ex apical localisation through a direct interaction between the FERM-binding motif of the Crb intracellular domain (ICD) and the N-terminal FERM domain of Ex. Crb is the only transmembrane protein that has been shown to directly bind Ex (Chen et al., 2010; Grzeschik et al., 2010; Ling et al., 2010; Robinson et al., 2010). Mutations in *crb* cause mislocalisation of Ex from the apical membrane to the cytoplasm, associated with an increase in Yki activity (Chen et al., 2010; Grzeschik et al., 2010; Ling et al., 2010; Robinson et al., 2010). In addition to promoting Ex localisation, Crb also regulates Ex levels by promoting its phosphorylation-dependent turnover. Overexpression of Crb stimulates Ex phosphorylation by Casein Kinase 1 (CK1) family kinases. This phosphorylation promotes the interaction of Ex with the F-box E3 ligase Supernumerary Limbs (Slmb) and results in Ex ubiquitination and proteasomal degradation (Fulford et al., 2019; Ribeiro et al., 2014).

**Figure 1:**
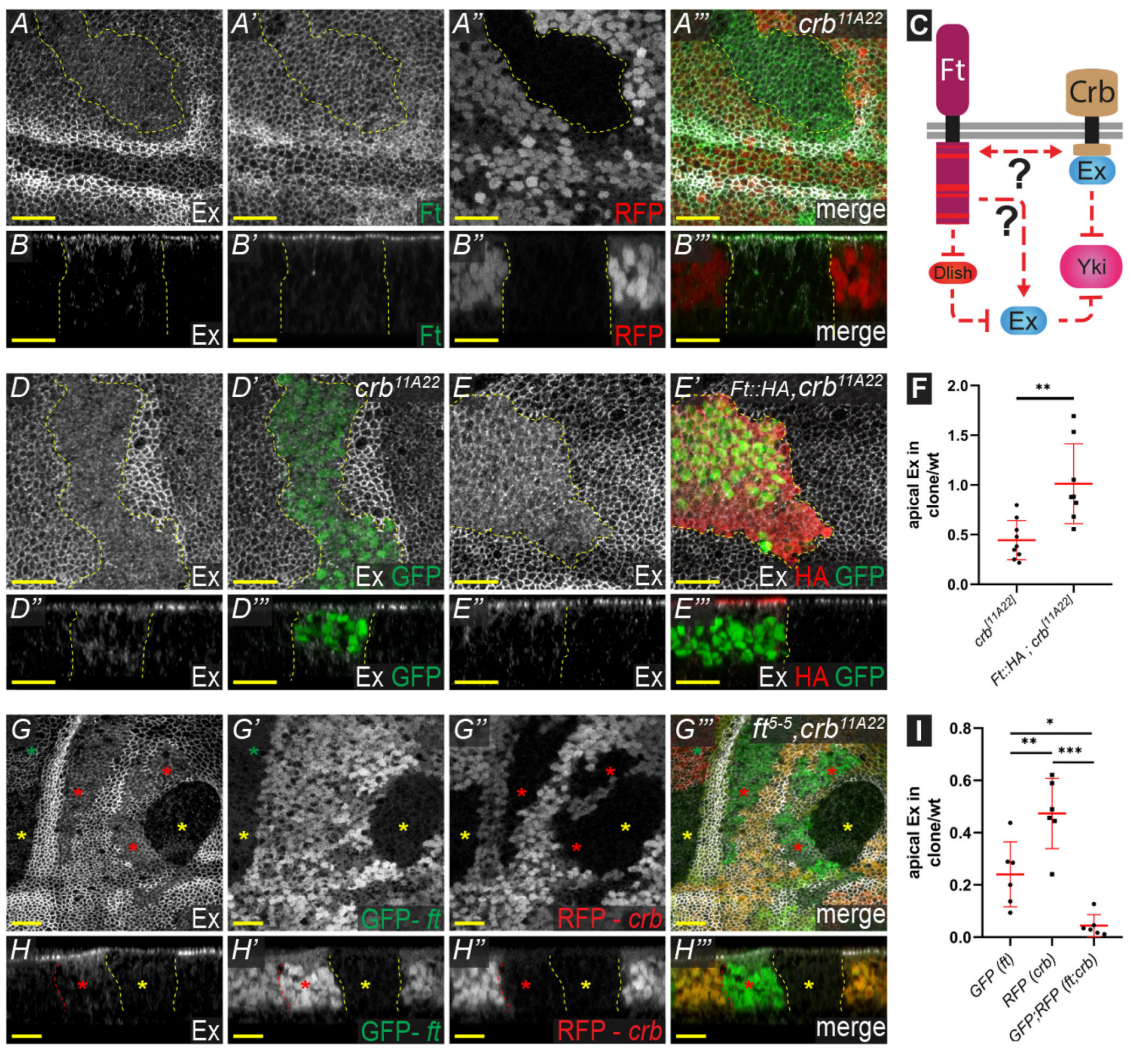
Ft and Crb regulate Ex independently **(A-B”’)** Loss of Crb does not affect Ft and residual Ex remains at the apical membrane. XY (A-A”’) and transverse (B-B”’) confocal micrographs of third instar wing imaginal discs containing *crb^11A22^* mutant clones (marked by absence of RFP), with Ex staining (grey in A, A”’, B and B”’), Ft staining (grey in A’ and B’ and green in A”’ and B”’) and RFP (grey in A” and B” and red in A”’ and B”’). **(C)** A model representing the current understanding of Ft and Crb dependent regulation of Ex. Red dashes within the Ft ICD represent the conserved regions A-F. **(D-E”’)** Overexpression of Ft within *crb* mutant tissue rescues apical Ex. XY (D-D’ and E-E’) and transverse (D”-D”’ and E”-E”’) confocal micrographs of third instar wing imaginal discs containing MARCM *crb^11A22^* clones without UAS-expression (D-D”’) or expressing UAS-Ft::HA (E-E”’). Clones are marked by GFP (green in D’ and D”’ and E’ and E”’) and are stained with Ex (grey) and Ft (visualised by HA staining, red in E’ and E”’). **(F)** Quantification of the ratio between apical Ex inside versus outside the MARCM clone normalised to the wildtype tissue. Data points represent an average of a single disc with the mean and standard deviation indicated. **P= 0.0018 using an unpaired T-test. **(G-H”’)** Loss of Crb and Ft have an additive effect causing dramatic loss of apical Ex. XY (G-G”’) and transverse (H-H”’) confocal micrographs of third instar wing imaginal discs containing *ft^5^-5* (marked by absence of GFP − grey in G’ and H’, green in G”’ and H”’ and by green asterisks) and *crb^11A22^* mutant clones (marked by absence of RFP − grey in G” and H”, red in G”’ and H”’ and by red asterisks), with Ex staining (grey in G, G”’, H and H”’). *ft^5^-5* is a remake of *ft^fd^* and is a null allele. Double mutant clones are marked by absence of GFP and RFP and by yellow asterisks. **(I)** Quantification of the ratio between apical Ex inside indicated clone verses outside the clone. All quantification was performed on the genotype used to create double clones. Data points represent an average of a single disc with the mean and standard deviation indicated. *P= 0.0177, **P= 0.0054 and ****P< 0.0001 using one-way ANOVA with a Tukey’s post-hoc test. All XY images are orientated as dorsal up and all transverse images are apical up. Clonal boundaries are marked by yellow dotted lines. Scale bars are 10 µm.

Ft is a giant atypical cadherin that localises to the apical junctions where it regulates Hippo signalling and PCP, in part through heterophilic interaction with the atypical cadherin Dachsous (Ds) (Blair and McNeill, 2018; Fulford and McNeill, 2019; Irvine and Harvey, 2015). Ft also regulates Ex localisation and levels (Bennett & Harvey, 2006; Cho et al., 2006; Silva et al., 2006), however if Fat regulates Ex directly is not known. The Ft ICD is crucial in implementing its biological function. Several structure-function studies have identified key regions within the ICD that mediate its signalling, including Hippo functional domains, and highly conserved regions A to F (Bossuyt et al., 2014; Matakatsu & Blair, 2012; Pan et al., 2013; Zhao et al., 2013) (FIG1C, 4A).

Ft inhibits growth in part by limiting levels and activity of the atypical myosin Dachs (Mao et al., 2006), which regulates growth by destabilising and sterically inhibiting Wts, thereby activating Yki function (Cho et al., 2006; Rauskolb et al., 2011; Vrabioiu & Struhl, 2015). Ft suppresses growth in concert with the CK1 kinase Discs overgrown (Dco) and the F-box E3 ubiquitin ligase Fbxl7. Dco phosphorylates the Ft ICD contributing to its activation (Cho et al., 2006; Feng & Irvine, 2009; Pan et al., 2013; Sopko et al., 2009), while Fbxl7 interacts with Ft and limits Dachs accumulation (Bosch et al., 2014; Rodrigues-Campos & Thompson, 2014).

The palmitoyltransferase Approximated (App) and the SH3 containing protein Dlish (also known as Vamana) antagonise Ft activity and promote Dachs activity. Single mutations of *dachs, app* or *dlish* have only mild undergrowth phenotypes alone, but can suppress the overgrowth induced by loss of *ft* (Mao et al., 2006; Matakatsu and Blair, 2008; Misra and Irvine, 2016; Zhang et al., 2016). App, Dlish and Dachs form a complex at the apical membrane, stabilising Dachs localisation and enhancing its activity (Matakatsu et al., 2017; Matakatsu and Blair, 2008; Misra and Irvine, 2016; Zhang et al., 2016). App can palmitoylate Dlish and regulate Dachs independently of its enzymatic activity (Matakatsu et al., 2017). App also palmitoylates Ft, antagonising the action of Dco (Matakatsu et al., 2017). The complex relationship between these proteins is thought to precisely tune Hippo pathway activity (Matakatsu et al., 2017).

Ft can regulate Ex localisation and levels through Dlish (Wang et al., 2019). Dlish can regulate Hippo signalling independently of Dachs by regulating Ex turnover (Wang et al., 2019). Dlish directly binds to the C-terminus of Ex, promoting the interaction between Ex and Slmb, thereby stimulating Ex degradation via the proteasome. This process is antagonised by Wts phosphorylation at Ex S1116, which stabilises Ex by protecting it from Slmb-mediated turnover (Zhang et al., 2015). C-terminal regulation of Ex by Slmb appears to be independent of the Crb-mediated regulation of the Ex N-terminus (Fulford et al., 2019). The E3 ubiquitin ligase Plenty of SH3s (POSH) has also been implicated in regulating Ex levels by binding to the Ex C-terminus and promoting its degradation (Ma et al., 2018), though this appears to be independent of Dlish (Wang et al., 2019).

As detailed above, Ex localisation, stability and activity are finely tuned by a complex molecular machinery. However, whether Ft regulation of Ex is direct, the role of Ds in these processes and the relationship between Ft and Crb to control Ex has remained unclear. Here we report that Ft promotes apical localisation of Ex by tethering it to the apical membrane, mediated by a direct interaction between the Ex FERM domain and the conserved E region of the Ft ICD. These processes occur independently of Crb. Using CRISPR, we determine that deletion of the conserved E region of Ft leads to wing overgrowth, reduced apical Ex localisation and increased levels of Dachs and Dlish. We show binding between the intracellular domains of Ft and Ds. Remarkably, we find that Ft can regulate Ex independently of Ds binding, as loss of *ds* upregulates Dachs/Dlish but does not downregulate Ex. This intricate regulation of Ex highlights its importance as an integrator of distinct polarity cues in the control of tissue growth.

## Results

### Ft and Crb independently regulate Ex levels and localisation

Loss of Crb results in the mislocalisation of Ex from the apical junction to the cytoplasm in imaginal disc epithelia (Chen et al., 2010; Grzeschik et al., 2010; Ling et al., 2010; Robinson et al., 2010). We confirmed Ex mislocalisation in *crb* clones, however, upon close examination we observed that a subset of Ex was still present at the apical membrane in the absence of Crb. Residual apical Ex was seen in *crb* null tissue using an Ex-specific antibody or a GFP-tagged knock-in Ex allele (Ex::GFP), and observed in clones of two different *crb* null alleles [FIG1A-B, suppFIG1A-B]. We confirmed both loss of Crb protein [suppFIG1C-D] and Ex antibody specificity [suppFIG1E]. These data indicate that there is a portion of Ex that localises to the apical membrane independently of Crb.

In addition to Crb, Ft regulates Ex localisation and levels [FIG1C] (Bennett & Harvey, 2006; Cho et al., 2006; Silva et al., 2006; Wang et al., 2019). We confirmed these data and further showed that Crb, Ft and Ex tightly colocalise [suppFIG1F-H], including within apical punctae where Ft has previously shown to localise (Ma et al., 2003a; Brittle et al., 2012; Hale et al., 2015) [suppFIG1I]. We also find loss of *ft* does not dramatically affect Crb levels [suppFIG1J]. Interestingly, the remaining apical Ex within *crb* mutant clones colocalises with Ft [FIG1A-B]. We tested the ability of Ft to contribute to Ex apical localisation by comparing *crb* null clones and *crb* null clones expressing full-length, HA-tagged Ft (Ft::HA). Notably, increasing levels of Ft within a *crb* clone significantly rescues apical Ex [FIG1D-F]. This increase in apical Ex is not accompanied by alterations in basal Ex [suppFIG1K]. suggesting an overall increase in Ex protein, consistent with previous studies (Bennett and Harvey, 2006; Silva et al., 2006; Wang et al., 2019). This indicates that Ft can promote Ex apical localisation and stability independently of Crb.

We next investigated whether Ft regulates Ex localisation independently of Crb by examining double mutant null clones in the wing disc. As *ft* and *crb* are on different chromosomes, FLP-FRT mediated recombination occurs independently, generating patches of tissue mutant for only *ft* or *crb*, as well as *ft;crb* double mutant tissue. In the single mutant tissue, as expected, loss of *crb* or *ft* results in a significant reduction in apical Ex [FIG1G-I]. Notably loss of *crb* additionally results in an increase in cytosolic Ex not seen in *ft* mutant clones, consistent with previous studies [FIG1H] (Chen et al., 2010; Ling et al., 2010; Robinson et al., 2010). Interestingly, *ft* mutation results in a greater loss of apical Ex than *crb* mutation [FIG1I]. In *ft;crb* clones there is a dramatic and near total loss of Ex when compared to either single mutant [FIG1G-I]. Thus, loss-of-function experiments also indicate that Ft and Crb regulate Ex independently.

### Ex directly binds to Ft

**Figure 2:**
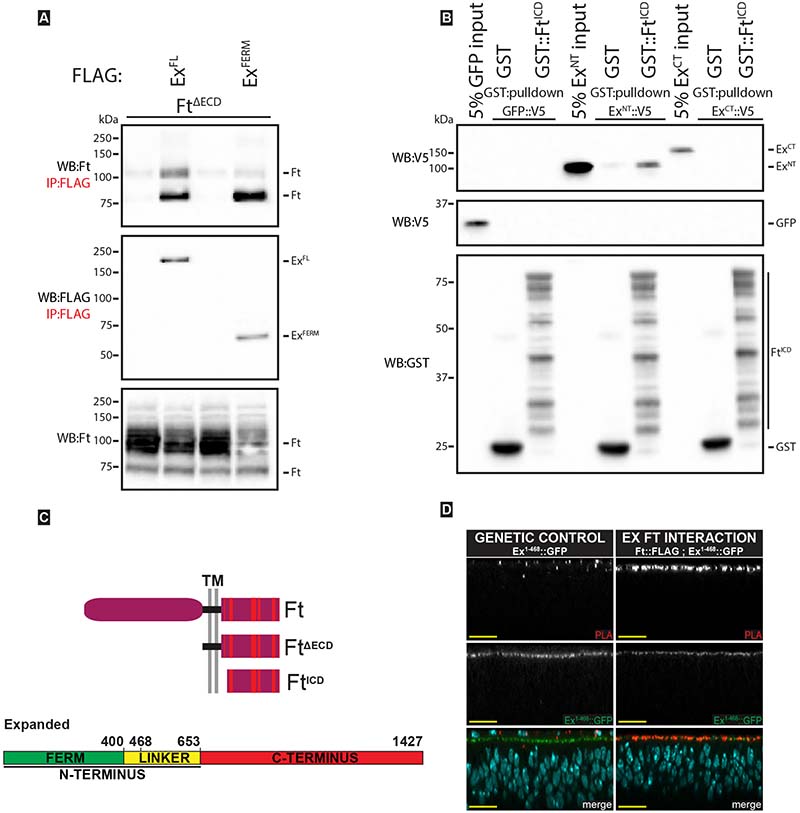
Ft and Ex directly bind *in vitro* and *in vivo* **(A)** Ft^ΔECD^ interacts with Ex^FL^ and Ex^FERM^. S2R+ cell expression and IP of FLAG-tagged Ex^FL^ or Ex^FERM^ in the presence of Ft^ΔECD^, compared to FLAG-bead controls. Ft presents as multiple bands due to proteolytic processing (Feng & Irvine, 2009; Sopko et al., 2009). **(B)** Ft^ICD^ directly binds Ex^NT^. *In vitro* transcribed and translated GFP as a control, Ex^NT^ and Ex^CT^ were incubated with bacterially expressed and purified GST alone or GST::Ft^ICD^ and subjected to GST-purification. The expression and presence of proteins was analysed by immunoblotting with the indicated antibodies. **(C)** Schematic representation of Ft, Ft^ΔECD^ and Ft^ICD^ and Ex protein. Red dashes within the Ft ICD represent the conserved regions A-F. **(D)** Ft and Ex interact at apical membrane *in vivo*. Transverse confocal micrographs of third instar imaginal discs expressing *ubi-Ex^1^-468::GFP* subjected to anti-FLAG and anti-GFP PLA. Genetic control expresses only *ubi-Ex^1-468^::GFP* with wildtype Ft, and the Ex::Ft interaction condition expresses *ft::FLAG* at the endogenous locus and *ubi-Ex^1^-468::GFP*. Ex^1^-468::GFP is observed by direct fluorescence of GFP (grey or green in merge), PLA signal (grey or red in merge) mark interaction loci and Hoechst (cyan in merge) marks nuclei. Sections are orientated apical up. Scale bars are 10 µm.

Both gain and loss of function studies indicate that Ft regulates Ex independently of Crb but does not address how Ft controls Ex localisation. To test if Ft physically associates with Ex, we co-expressed a form of Ft (Ft^ΔECD^), which rescues growth defects caused by null mutations of *ft* (Matakatsu & Blair, 2006) with full-length (Ex^FL^) or the N-terminal FERM domain of Ex (Ex^FERM^) in S2R+ cells (see [FIG2C] for Ex domains and Ft structure). Both Ex^FL^ and Ex^FERM^ robustly co-immunoprecipitated (co-IP) Ft^ΔECD^ [FIG2A], indicating that Ft and Ex can form a biochemical complex.

To determine if the interaction between Ft and Ex is direct, we performed a pulldown assay between bacterially expressed and purified GST-Ft^ICD^ and *in vitro* translated N-terminal (Ex^NT^) and C-terminal Ex (Ex^CT^) along with GFP as a negative control. Compared to GST alone, GST-Ft^ICD^ significantly binds to Ex^NT^ but not Ex^CT^ [FIG2B]. We also tested Ex^1^-468 and found this smaller fragment of Ex also directly binds Ft^ICD^ [suppFIG2A]. These data reveal Ft binds directly to Ex. These data also indicate the Fat-Ex interaction occurs via the N-terminal Ex FERM domain.

Next, we investigated if the Ft-Ex interaction occurs *in vivo* by performing an *in-situ* proximity ligation assay (PLA), an immuno-PCR-based technique producing a positive fluorescent signal when two antibody epitopes are no further than approximately 40 nm apart and presumably interacting directly (Alam, 2018). In addition, PLA provides data on the localisation of protein interactions. To perform this technique, we used the FERM-domain containing Ex^1-468^ truncation, driven by the *ubiquitin 63E* promoter (ubi-Ex^1^-468::GFP) (Fulford et al., 2019), which colocalises with Ft [suppFIG2B] and a C-terminal FLAG-tagged Ft knock-in allele we generated for this study. Consistent with our biochemical data, we observed PLA signal colocalising with Ex signal at the apical membrane in in XZ [FIG2D] and XY [suppFIG2C] sections of imaginal wing discs, supporting a direct interaction *in vivo* between Ft and Ex.

Dlish has recently been shown to directly bind the C-terminus of Ex and regulate its turnover downstream of Ft (Wang et al., 2019). Dlish and Ft have been shown to interact via co-IP from cultured cells (Misra and Irvine, 2016; Zhang et al., 2016) but it is unclear if this interaction is direct. We therefore tested whether Dlish could also bind FtICD through an *in vitro* binding assay similarly to Ex but could see no evidence of direct binding [suppFIG2D]. Taken together, these data support the hypothesis that the Ex FERM domain binds directly to Ft in vivo at the apical membrane and suggest a model where Ex biochemically links Ft and Dlish.

### Ft contains two Ex interaction domains

**Figure 3:**
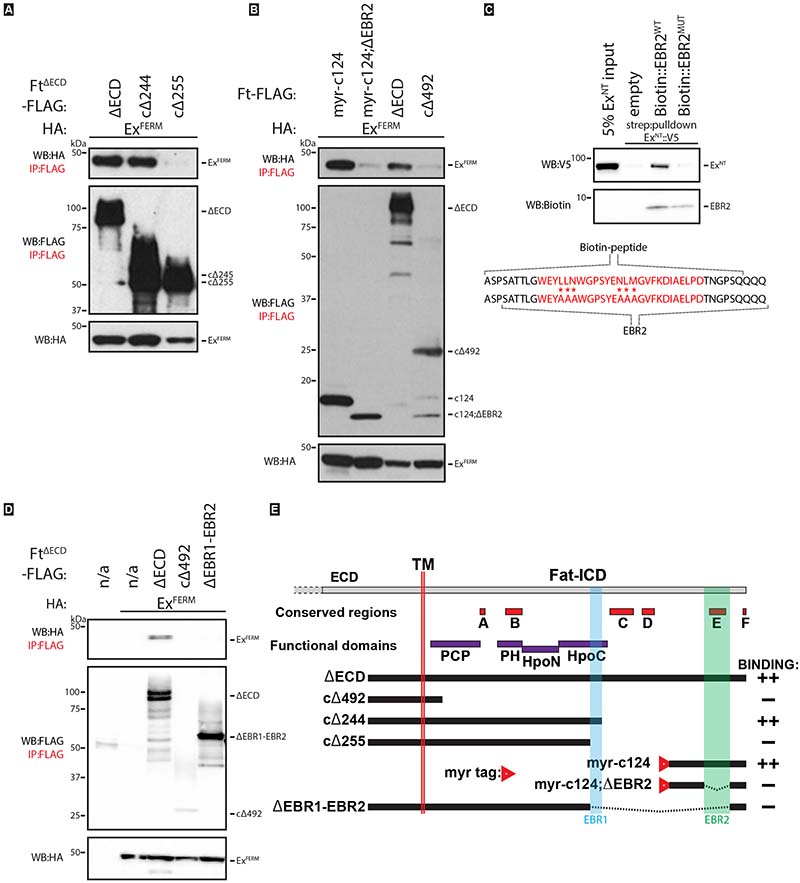
Identification of Expanded Binding Regions within the Ft-ICD **(A)** Identification of Expanded Binding Region (EBR) 1 in the Ft-ICD. HEK293 cell expression and IP of indicated FLAG-tagged Ft^ΔECD^ constructs in the presence of Ex^FERM^. **(B)** Identification of Expanded Binding Region (EBR) 2 in the Ft-ICD. HEK293 cell expression and IP of indicated FLAG-tagged Ft^ΔECD^ or FLAG- and myristoyl-tagged constructs in the presence of Ex^FERM^. **(C)** EBR2 directly binds Ex^NT^. *In vitro* transcribed and translated Ex^NT^ was incubated alone or with Biotin-tagged EBR2^WT^ or EBR2^MUT^ peptide (sequences indicated − mutant peptide containing 6 alanine substitutions) and subjected to streptavidin-purification. EBR2 sequence defined by co-IP and the conserved-E region (highlighted in red) are also indicated. **(D)** Identification of the Expanded interacting region of the Ft-ICD. HEK293 cell expression and IP of indicated FLAG-tagged Ft^ΔECD^ constructs in the presence of Ex^FERM^ compared to FLAG-bead controls. The expression and presence of proteins was analysed by immunoblotting with the indicated antibodies. Ft presents as multiple bands due to proteolytic processing (Feng & Irvine, 2009; Sopko et al., 2009). **(E)** Graphical scheme highlighting the Ft constructs used in figure 3. In addition, the transmembrane domain (TM), EBR1, EBR2 and established conserved and function domains of the Ft-ICD are depicted. In binding column: ‘++’ denotes constructs that interact strongly to Ex^FERM^ and ‘-’ denotes no interaction with Ex^FERM^.

**Figure 4:**
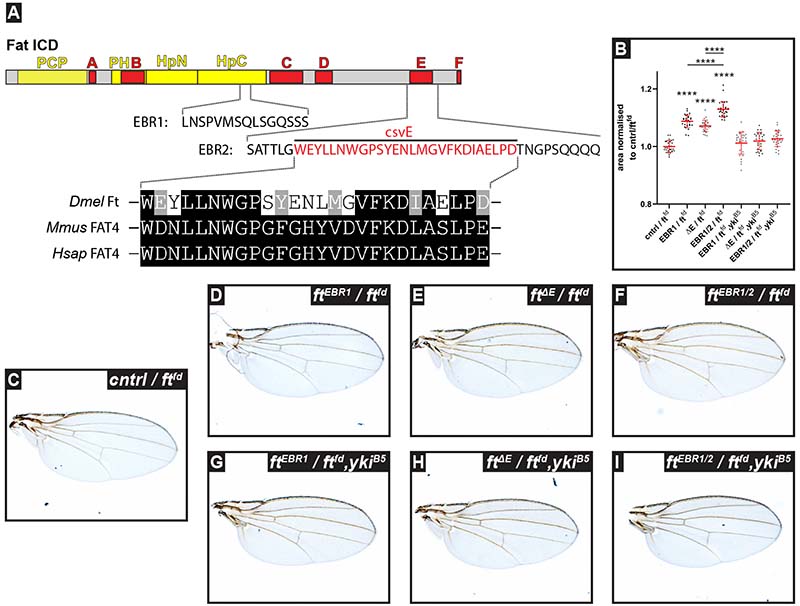
EBRs are required *in vivo* for regulation of tissue growth **(A)** Schematic representation of the Ft-ICD highlighting functional and conserved domains. EBR1, EBR2 and conserved-E location and amino acid sequence are depicted, in addition to the conservation of the conserved-E region (in red) between flies, mice and humans. EBR1 and EBR1/2 flies have the indicated sequences removed and have a C-terminal 3x FLAG. csvE has the indicated sequence (highlighted in red) replaced with a 3x FLAG. **(B)** Quantification of adult wing size with haploinsufficiency for *ft* or *ft, yki*. Data are normalised against the mean of the *cntrl* / *ft^fd^* (*ft::FLAG / ft^fd^*). All EBR flies are overgrown, with ft^EBR1/2^ causing additive overgrowth. Haploinsufficiency of *ft, yki* rescues overgrowth (**** significance for ft^EBR1^, ft^ΔE^ and ft^EBR1/2^ compared to the respective genotypes haploinsufficient for *ft* alone). Data points indicate an individual wing with mean and standard deviation represented. ****P<0.0001 using one-way ANOVA with a Tukey’s post-hoc test compared to *cnt*rl / *ft^fd^* or between indicated genotypes. **(C-I)** EBR deletion causes overgrowth and is rescued by haploinsufficiency of *yki*. Compared to cntrl wings - ft::FLAG (C) loss of *ft^EBR1^* (D), *ft^ΔE^* (E) and *ft^EBR1/2^* (F) cause overgrowth when over the null *ft^fd^* allele. Additional loss of one copy of *yki* rescues overgrowth of all indicated genotypes *ft^EBR1^* (G), *ft^ΔE^* (H) and *ft^EBR1/2^* (I).

Our data indicates that the Ft ICD binds directly to the Ex FERM [FIG2B, suppFIG2A]. To determine which region of Ft interacts with the FERM domain, we performed co-IPs using truncated and/or internally deleted Ft^ΔECD^ constructs in HEK293 cells (see FIG3E and suppFIG3C). Significantly, Ft^c^Δ244 (removing 244 residues from the C-terminus of Ft^ΔECD^) can effectively co-IP Ex^FERM^, whereas removing 255 residues from the C-terminus of Ft^ΔECD^ (Ft^c^Δ255) completely abolished the interaction with Ex^FERM^ [FIG3A]. These data indicate the amino acids between residues 255-244 from the Ft C-terminus are needed to interact with Ex^FERM^, defining Expanded Binding Region 1 (EBR1). Interestingly, this region is within the Hippo activating domain of the Ft ICD as defined in several structure-function analyses of Ft (Bossuyt et al., 2014; Matakatsu & Blair, 2012; Pan et al., 2013; Zhao et al., 2013) [FIG3E, FIG4A].

Further deletion and co-IP analyses revealed the existence of a second Ex binding site in the C-terminus of Ft [suppFIG3A]. This binding site includes the E and F domains (defined in (Pan et al., 2013)), conserved across multiple species including human FAT4. To confirm this interaction, we generated a construct containing the last 124 residues of Ft tagged with a myristoylation sequence targeting it to cell membranes (Ft^myr-c124^). Ft^myr-c124^ can interact with Ex^FERM^ [FIG3B] indicating that the C-terminal 124 residues of Ft can bind Ex. To narrow down the C-terminal binding domain further, we created an internal deletion within Ft^myr^-c124, which removes a Ft ICD fragment containing the conserved E region (Ft^myr^-c124;ΔEBR2). This deletion abolished the interaction between Ft and Ex [FIG3B] indicating an Ex-binding region lies between the C-terminal residues 64-25, which we named EBR2 [FIG3E, FIG4A]. Interestingly, neither EBR1 nor EBR2 contain known FERM binding motifs (Gunn-Moore et al., 2006; Ling et al., 2010).

We confirmed a direct interaction between Ft conserved E region and Ex by generating a biotin-tagged peptide − called EBR2^WT^ − and performed a streptavidin-pulldown with recombinant Ex^NT^ [FIG3C]. In contrast, a biotin-tagged EBR^MUT^ peptide, with 6 residues mutated to alanine was unable to interact with Ex^NT^, highlighting the importance of the conserved E region in the binding to Ex [FIG3C].

To explore whether additional conserved regions affect the Ft-Ex interaction we generated Ft^ΔECD^ constructs from EBR1 through to the conserved D region (Ft^cΔ492-256;cΔ153^), removing the C region (Ft^cΔ492-256;ΔC;cΔ153^), D region (Ft^cΔ492-256;ΔD-CT^) or both (Ft^cΔ492-256;ΔC;ΔD-CT^). Interestingly, we found that loss of either of these conserved regions reduced Ft-Ex interaction, and removal of both completely abolished it, although these constructs contain EBR1 [suppFIG3B]. These data show that the conserved C and D regions contribute to the Ft-Ex interaction. Deleting the entire region from EBR1 to EBR2 (Ft^ΔEBR1-EBR2^) abrogates Ex binding [FIG3D].

### Ex binding regions of the Ft ICD are required *in vivo* for regulation of tissue growth

Having mapped the regions of the Ft ICD that interact with Ex, we next investigated their biological significance *in vivo*. We used CRISPR to delete EBR1 (Ft^EBR1^) and both EBR1 and EBR2 (Ft^EBR1/2^), from the endogenous *ft* locus, and added a 3xFLAG tag to the C-terminus. In addition, we used CRISPR to remove the conserved E region (largely overlapping EBR2), and replaced it with a 3xFLAG tag (Ft^ΔE^) [FIG4A]. Immunoblot and clonal analysis indicated deletion of these regions in Ft^EBR1^, Ft^ΔE^ and Ft^EBR1/2^ did not affect levels of Ft protein, nor perturb Ft localisation [suppFIG4A-D]. To account for CRISPR-induced second site hits, we performed trans-heterozygous analysis of two independent lines for each mutation, which produced viable and fertile animals for all genotypes [suppFIG4E-I] despite significant pupal lethality in Ft^ΔE^ and Ft^EBR1/2^ [suppFIG4J]. Due to the pupal lethality seen in Ft^ΔE^ and Ft^EBR1/2^, we analysed wing growth phenotypes as trans-heterozygous to the *ft^fd^* null allele. Interestingly, in this sensitised background, all three genotypes produced overgrown wings compared to the ft::FLAG control, with Ft^EBR1/2^ producing overgrowth in excess of either Ft^ERB1^ or Ft^ΔE^ [FIG4B-F].

Further analysis of the EBR trans-heterozygous flies (independent lines of the same mutation) confirmed Ft^EBR1^ causes wing overgrowth, consistent with EBR1 residing in the HpoC functional domain that affects Hippo signalling [suppFIG4E-F,K]. However, Ft^ΔE^ flies were not overgrown [suppFIG4G,K], and Ft^EBR1/2^ wings were mostly undergrown (class 1) [suppFIG4H,K], with a distinct subpopulation of Ft^EBR1/2^ flies (class 2) that were significantly larger than the controls [suppFIG4I,K]. The reason for this phenotypic separation remains unclear but could be due to developmental defects causing the significant pupal lethality observed (suppFIG4J). Nevertheless, the population of Ft^EBR1/2^ flies that were overgrown (class 2) all had rounded wings with cross-vein defects [suppFIG4I], which was also observed in Ft^ΔE^ [suppFIG4G], indicating the E region affects wing shape. We calculated the ratio of length to width to measure the wing roundness, a typical phenotype of Ft/Ds pathway mutants (Mao et al., 2006; Matakatsu & Blair, 2006). Both Ft^EBR1^ and Ft^ΔE^ had significantly rounder wings than controls [suppFIG4L]. As with wing size, Ft^EBR1/2^ separated into distinct populations, with the class 2 subpopulation generating wings that were significantly rounder than the control [suppFIG4L].

To assess whether the overgrowth was the result of excessive Yki activity, we analysed adult wing phenotypes in a *ft, yki* haplo-insufficient background. Importantly, overgrowth of EBR mutant flies was suppressed when one copy of Yki was removed, consistent with the hypothesis that overgrowth due to loss of Ex binding to Ft is Hippo pathway dependent [FIG4B-I].

### Conserved E region/EBR2 is required to regulate Ex, Dachs and Dlish *in vivo*

**Figure 5:**
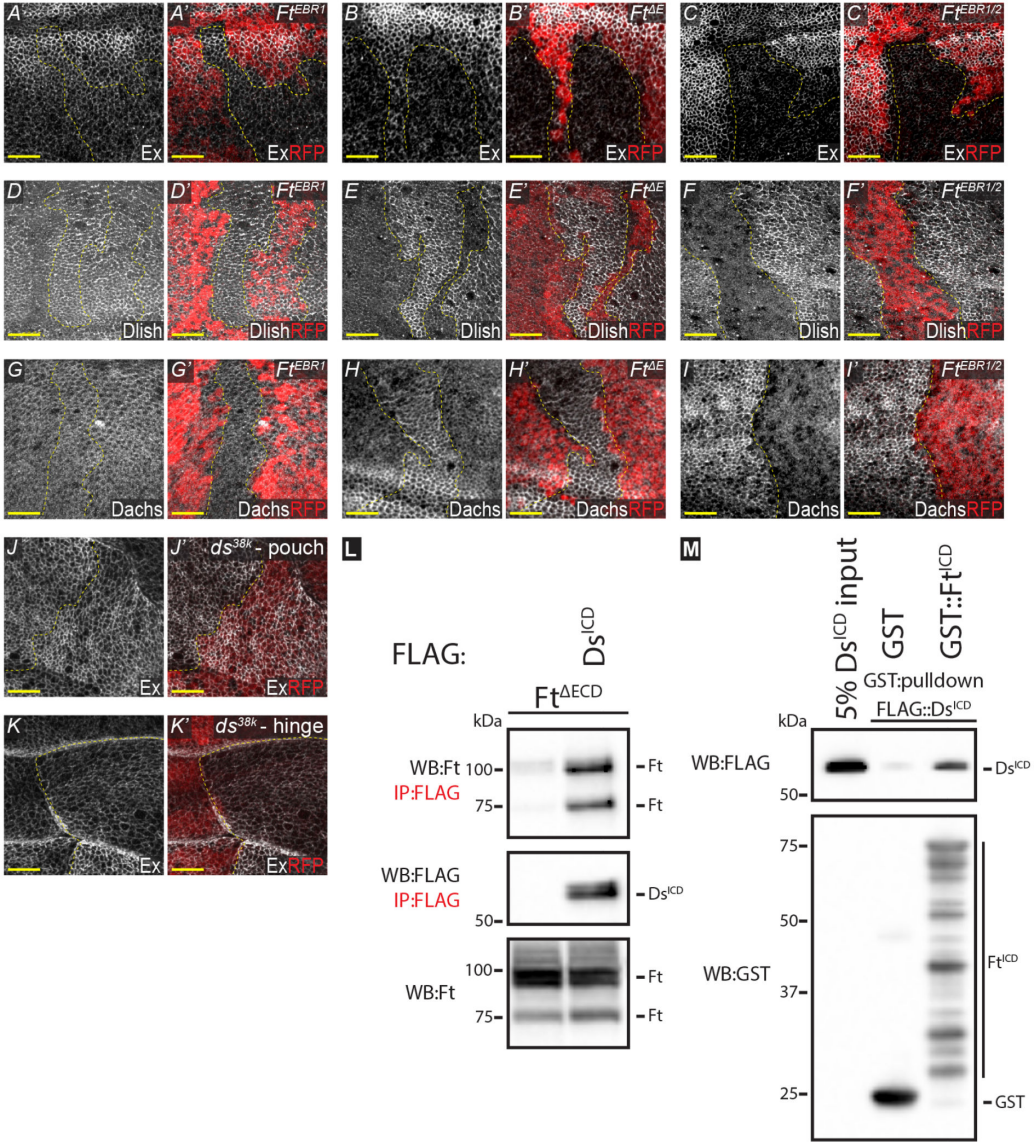
EBR clonal analysis and Ft-Ds interaction **(A-C’)** *ft^ΔE^* and *ft^EBR1/2^* cause a reduction of apical Ex, unlike *ft^EBR1^*. XY confocal micrographs third instar wing imaginal discs containing *ft^EBR1^* (A-A’), *ft^ΔE^* (B-B’) and *ft^EBR1/2^* (C-C’) mutant clones (marked by absence of RFP shown in red) with Ex staining (shown in grey). **(D-I’)** *ft^ΔE^* and *ft^EBR1/2^* cause an increase of apical Dlish and Dachs, unlike *ft^EBR1^*. XY confocal micrographs third instar wing imaginal discs containing *ft^EBR1^* (D-D’ and G-G’), *ft^ΔE^* (E-E’ and H-H’) and *ft^EBR1/2^* (F-F’ and I-I’) mutant clones (marked by absence of RFP shown in red) with Dlish (D-F’) or Dachs (G-I’) staining (shown in grey). **(J-K’)** Loss of *ds* subtly increases Ex. XY confocal micrographs of the same third instar wing imaginal disc showing the pouch (J-J’) and the hinge (K-K’) containing *ds^38K^* mutant clones (marked by absence of RFP shown in red) with Ex staining (shown in grey). All XY images are orientated as dorsal up. Clonal boundaries are marked by yellow dotted lines. Scale bars are 10 µm. **(L)** Ft^ΔECD^ interacts with Ds^ICD^. S2R+ cell expression and IP of FLAG-tagged Ds^ICD^ in the presence of Ft^ΔECD^, compared to FLAG-bead controls. Ft presents as multiple bands due to proteolytic processing (Feng & Irvine, 2009; Sopko et al., 2009). **(M)** Ft^ICD^ directly binds Ds^ICD^. *In vitro* transcribed and translated Ds^ICD^ was incubated with bacterially expressed and purified GST alone or GST::Ft^ICD^ and subjected to GST-purification. The expression and presence of proteins was analysed by immunoblotting with the indicated antibodies.

To mechanistically understand the effects of loss of Ex binding regions on the Hippo pathway, we generated mitotic clones of our new alleles in wing discs and stained for Ex, as well as for Dlish and Dachs, critical mediators of Fat-Hippo signalling (Mao et al., 2006; Misra and Irvine, 2016; Zhang et al., 2016). Ft restricts Dlish and Dachs apical localisation, and Dlish stimulates Ex degradation (Wang et al., 2019). Loss of Ft leads to reduction of Ex [FIGsupp1F] and increased expression of Dachs and Dlish as previously reported (Bennett and Harvey, 2006; Mao et al., 2006; Misra and Irvine, 2016; Silva et al., 2006; Zhang et al., 2016). Clones of cells homozygous for *ft^EBR1^* caused no change in levels or distribution of Ex, Dachs or Dlish [FIG5A,D,G], indicating this region is not critical for regulation of these proteins. Importantly, clones of *ft^ΔE^* or *ft^EBR1/2^* showed a reduction in apical Ex [Fig5B-C], and a dramatic increase in both Dlish and Dachs [FIG5E-F,H-I]. Together, these data indicate that conserved region E is essential for restricting Dlish and Dachs and stabilising Ex *in vivo.* Interestingly*, ft^EBR1/2^* clones appear to cause a greater loss of apical Ex than *ft^ΔE^*, which may indicate a contribution of EBR1 [Fig4A].

As Crb and Dlish both regulate Ex, we wondered whether loss of Crb alters Dachs/Dlish, and therefore regulate Ex through these proteins. Neither Dachs nor Dlish was altered in *crb* clones [suppFIG5A-B], indicating that Ft and Crb independently regulate Ex. As Dlish binds to the Ex C-terminus, these data are also consistent with previous studies showing the Slmb-mediated regulation of the Ex C-terminus is independent of Crb (Fulford et al., 2019).

Loss of Fbxl7 increases apical Dachs (Bosch et al., 2014; Rodrigues-Campos & Thompson, 2014). As increased apical Dachs is associated with a concurrent increase in apical Dlish, we investigated whether loss of Fbxl7 could also regulate Ex. Knockdown of Fbxl7 did not alter levels of endogenous Ex observed through staining, or levels of the ubi-Ex^1^-468::GFP construct that does not respond to changes in Yki transcription [suppFIG5C-D]. These data suggest Fbxl7 does not regulate Ex.

### Increased Dachs and Dlish do not reduce Ex levels in Ds mutants

Ds is the only known ligand of Ft, and spatial gradients of Ds expression are thought to regulate Ft activity (Strutt & Strutt, 2021). Null mutations of *ft* or *ds* result in increased levels of apical Dachs and Dlish and Hippo pathway dependent overgrowth (Brittle et al., 2012; Misra and Irvine, 2016; Zhang et al., 2016). We confirmed Ds repression of Dachs and Dlish, generating clones of *ds^38k^*, a protein null [suppFIG5E]. *ds* clones display clear increases in both Dachs and Dlish [suppFIG5F-G]. We also stained *ds* clones for Crb and observed no change [suppFIG5H].

Given the current model where increased levels of Dlish-Ex complex stimulates Ex degradation (Wang et al., 2019), loss of Ds should lead to reduced Ex. We therefore investigated whether Ds regulates Ex. Remarkably, *ds* loss in wing discs did not decrease Ex levels and often resulted in a subtle increase in Ex, particularly in the imaginal disc hinge region, where Ds expression is highest [FIG5J-K]. This is in stark contrast to *ft* mutant clones, which dramatically decrease Ex [suppFIG1F]. Loss of *ds* has a limited effect on Ft staining in the wing pouch, with subtly diffuse but still apical Ft [suppFIG5I] (Strutt and Strutt, 2002; Mao et al., 2009). These surprising data indicate that, despite Ds regulating Dachs and Dlish, there is no reciprocal reduction in Ex, and further indicate that levels of Dachs/Dlish can be uncoupled from levels of Ex.

Our finding that Ft and Ds both regulate Dachs and Dlish levels, yet loss of Ds does not cause a reduction in Ex, prompted us to investigate whether Ds and Ex could bind. However, we found no evidence of a direct interaction between Ds and Ex^NT^ or Ex^CT^, consistent with Ft regulation of Ex being independent of Ds [FIGsupp5J]. As Ds and Dlish interact through co-IP (Misra and Irvine, 2016; Zhang et al., 2016), we tested if they could directly bind, but found no interaction [FIGsupp5K] suggesting these proteins likely need an intermediary to interact.

Although representations of Ft and Ds emphasize asymmetric distribution of these molecules, co-staining reveals that there is substantial overlap in Ft and Ds staining at cell membranes (Ma et al., 2003b). In addition, Ds puncta visualised by immunofluorescence are stabilised by the presence of Ft (Hale et al., 2015), and genetic data suggest that Ft and Ds may interact in *cis* (within cells) as well as in *trans* (Sharma & McNeill, 2013).

We therefore tested if Ft and Ds could interact independently of their previously documented extracellular domain interactions by performing co-IP of Ft^ΔECD^ with Ds^ICD^ in S2R+ cells. Ft^ΔECD^ removes most of the extracellular domain, including all cadherin repeats, EGF-like domains and Laminin-G domains, and retains the transmembrane domain, and the full ICD (Matakatsu & Blair, 2006). Ds^ICD^ removes the entire extracellular and transmembrane domains, and only retains the intracellular domain. Remarkably, Ds-ICD can co-immunoprecipitate with Ft^ΔECD^ [Fig5L], indicating that Ft and Ds can form a complex mediated by their cytoplasmic domains.

The co-IP of Ft and Ds mediated by their intracellular domains could be direct, or via intermediary proteins. We tested if this interaction was direct through a binding assay between GST-Ft^ICD^ and *in vitro* translated Ds^ICD^ and observed binding between the ICDs of these proteins [Fig5M]. These data indicate *cis* interactions between Ft and Ds within the cytoplasm can be mediated via their ICDs. Thus, Ft and Ds can interact both across cell borders via their extracellular cadherin repeats, and within cells via their intracellular domains. These intracellular interactions imply that complexes that are independently formed on Ft and Ds can be brought together, and this cross-regulation has the potential for regulating Hippo or PCP activity.

## Discussion

Ex is a critical nexus of Hippo signalling and is highly regulated, with its levels and localisation being controlled by two transmembrane proteins, Crb and Ft (Bennett and Harvey, 2006; Ling et al., 2010; Fulford et al., 2019; Ribeiro et al., 2014; Silva et al., 2006; Wang et al., 2019). Cell-cell interactions are a key aspect of Hippo pathway upstream regulation that is thought to underpin their role in maintaining tissue homeostasis (Fulford et al., 2018; Misra and Irvine, 2018; Zheng and Pan, 2019). Previously, Crb was the only transmembrane protein known to directly bind and regulate Ex function (Ling et al., 2010), and has been established as an apical hub of Hippo signalling (Genevet & Tapon, 2011; Su et al., 2017; Sun et al., 2015). Here we show that both Crb and Ft directly bind to Ex, and regulate Ex independently. Loss of Ft in the absence of Crb further reduces apical Ex localisation and overexpression of Ft in *crb* mutant tissue is sufficient to rescue apical Ex levels, consistent with increased Ex protein [FIG1D-I, suppFIG1K]. Meanwhile, loss of *crb* does not affect Dachs or Dlish, which regulate Ex downstream of Ft [suppFIG5A-B]. These data demonstrate the ability of Ft to recruit and stabilise Ex apically, independently of Crb. We observe residual apical Ex when both *crb* and *ft* are lost [FIG1G-H] suggesting there are additional mechanisms to regulate apical Ex, such as Ex interaction with apical Spectrins (Fletcher et al., 2015).

We show Ft directly binds to the Ex FERM domain at the apical membrane [FIG2B,D, supp2A,C], occurring through a direct interaction with the Ft conserved E region [FIG3C]. Removal of the EBRs leads to wing overgrowth, although the extent of this overgrowth depends on the genetic background [FIG4, suppFIG4]. Haploinsufficiency for *yki* rescues EBR mutant wing overgrowth [FIG4B-I] and loss of conserved region E alters the localisation of the Hippo pathway regulators Ex, Dachs and Dlish [FIG5A-I]. Combined, these data indicate that this conserved region regulates Hippo signalling, and that Ft interaction with Ex provides a Crb-independent hub for Hippo signalling at the apical membrane.

**Figure 6:**
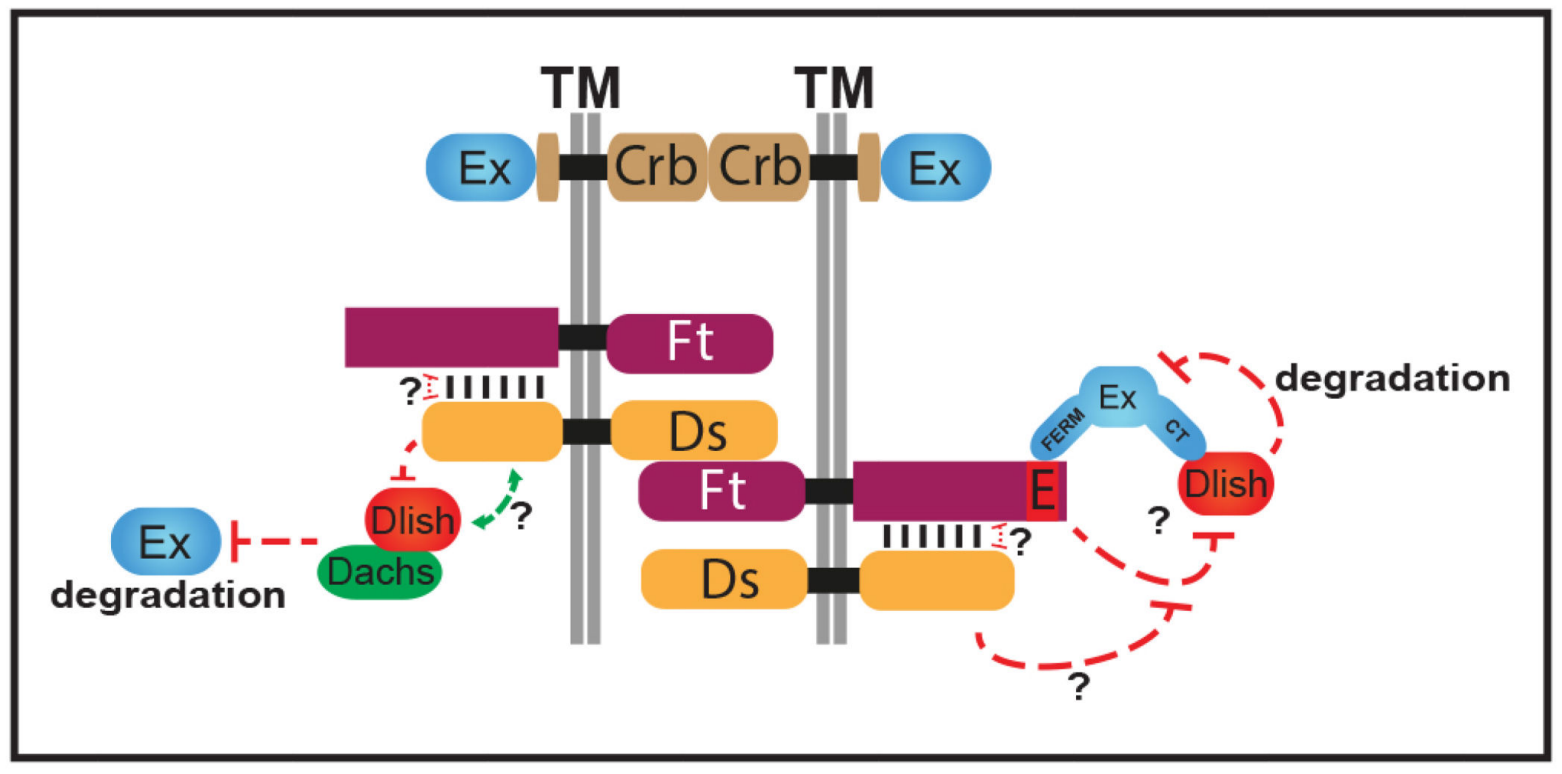
A model of Ft-mediated regulation of Ex. Graphical illustration of findings (not to scale). In wildtype conditions, Ft regulates Ex independently of Crb and there is a homeostasis of Ex levels. Ft promotes apical localisation of Ex. Degradation of Ex is stimulated by Dlish, which is balanced by Ft-mediated inhibition of Dlish. Loss of Ds increases the amount of apical Dlish available to interact with and degrade Ex, however this is counteracted by Ft actively inhibiting this process. Upon loss of Ft, or the conserved E region responsible for Ft-Ex interaction, Dlish is derepressed, increasing Ex-degradation. Ft and Ds *cis* interaction may act to antagonise Ft-mediated inhibition of Dlish, which may also promote mutual antagonism between Ft and Ds to support PCP.

While several Hippo pathway proteins have been identified as interacting with Ft (App, Dco, Dlish, Ds, FbxL7, Fj and Lft), or are regulated by Ft, so far none have been identified as a direct intracellular binding partner (Bosch et al., 2014; Brittle et al., 2010; Feng and Irvine, 2009; Ishikawa et al., 2008; Mao et al., 2009; Matakatsu et al., 2017; Sopko et al., 2009; Matakatsu and Blair, 2004; Misra and Irvine, 2016; Simon et al., 2010; Zhang et al., 2016). We show here that Ex binds directly to Ft. Ex also binds directly to Dlish, which promotes Slmb-dependent proteasomal degradation of Ex (Wang et al., 2019). Ex-Dlish binding occurs through the Ex C-terminus, whereas Ft binds to the N-terminal Ex FERM domain [FIG2B]. The region of the Ft ICD which interacts with Dlish (Zhang et al., 2016) overlaps with the conserved E region that binds Ex. Therefore, Dlish may interact with Ft via Ex. In the simplest model, a Ft-Ex-Dlish complex may conformationally inhibit Dlish, sequestering it away from Ex and preventing Ex degradation [FIG6]. Alternatively, the Ft-Ex-Dlish complex could inhibit the ability of App to palmitoylate Ft and/or Dlish (Matakatsu et al., 2017; Zhang et al., 2016). In this case, the Ex-Ft interaction would promote Ft signalling and limit the apical localisation of Dlish and Dachs, therefore inhibiting Dlish-dependent Ex degradation. Moreover, App antagonises activating phosphorylation of Ft by the CK1 kinase Dco (Matakatsu et al., 2017).

Unexpectedly, although loss of Ds results in increased Dachs/Dlish, Ex levels do not decrease [FIG5J-K, suppFIG5F-G]. We hypothesize that increased Dachs/Dlish levels are unable to stimulate Ex degradation in *ds* mutant cells because intact Ft can still bind Ex, protecting it from Dlish-dependent degradation [FIG6]. This is consistent with the ability of Ft to suppress growth independently of Ds (Matakatsu & Blair, 2006). Together our data shows Ft both directly contributes to apical localisation of Ex and promotes Ex stability, ensuring consistent levels of Ex protein, and Hippo pathway homeostasis.

We also discovered that in addition to their known extracellular *trans* interaction (Brittle et al., 2010; Matakatsu and Blair, 2004; Simon et al., 2010), the Ft and Ds ICDs interact directly via their cytoplasmic domains [FIG5L-M]. Previous studies have shown that although loss of Ds promotes growth, the Ds ICD itself can promote growth. This may occur through interaction with Dachs and Dlish to promote their activity (Bosveld et al., 2012; Misra and Irvine, 2016; Zhang et al., 2016) and through the phosphorylation and inhibition of Wts by the Minibrain kinase (Degoutin et al., 2013).

The subtle increase in Ex within *ds* clones [FIG5J-K] despite the strong increase in Dachs and Dlish suggests another potential mechanism by which the Ds ICD can promote growth − by antagonising Ft to promote Ex degradation. This could be influenced by the interaction of the Ft-Ds ICDs and suggests binding, in *trans* and *cis*, may modulate the interaction of Ft-Ex and Hippo signalling more broadly [FIG6].

Ft-Ex binding is dependent on regions that are highly conserved in Ft orthologues, including in mammals [FIG4A]. Interestingly, in mammals, Fat4 and Crb3 both regulate a functional orthologue of Ex, Amot. In mammalian cells, Amot binds to the Crb3 complex to regulate YAP/TAZ activity and, in the heart, Fat4 binds to Amotl1 to mediate YAP1 nuclear exclusion (Ragni et al., 2017; Varelas et al., 2010). It will be interesting to see whether the conserved regions of Fat4 contribute to its interaction with Amotl1 or other FERM domain orthologues of Ex and regulate YAP signalling.

## Materials and Methods

### *Drosophila* genetics and genotypes

*Ex::GFP* and *ft^5^-5* were generated by CRISPR/Cas9-mediated gene editing. *Ex::GFP* contains a C-terminal GFP tag. The *ex* genomic locus was cut near the stop codon by Cas9 guided by a gRNA (sequence: ATTAGCTTGTCGAGTCTAGC) and repaired from a co-injected plasmid template containing homologous sequence from the *ex* locus (2.4 kb upstream and 2.0 kb downstream of the stop codon), in which the eGFP coding sequence had been inserted immediately before the ex stop codon. *ft^5^-5* is a remake of the *ft^fd^* allele. The entire Ft locus was sequenced in wildtype *yw* and mutant *ft^fd^* flies, which identified a single nucleotide mutation in Tyr982 (TAT>TAA) generating a premature stop codon in the first exon. This mutation was re-generated using Cas9 guided by gRNA (sequence: GGGATGCGGGCGTGAATAGT) and repaired from a co-injected plasmid template containing homologous sequence (1.3 kb upstream and 1.3kb downstream of the *ft^fd^* mutation site) incorporating site directed mutagenesis to generate the T>A mutation. Progeny were genotyped and validated by sequencing.

*ft::FLAG*, *ft^EBR1^::FLAG* and *ft^EBR1/2^::FLAG*, all with C-terminal 3x FLAG tags and *ft^ΔE^::FLAG* with the conserved E region replaced by a 3x FLAG tag were generated by CRISPR/Cas9-mediated gene editing performed by GenetiVision. Sequences removed or replaced are indicated in [FIG4A]. Two independently generated lines for each genotype were analysed: *ft::FLAG (2)* and *(5), ft^EBR1^::FLAG (1-1)* and *(3-2)*, *ft^ΔE^::FLAG (1-2)* and *(5-2)* and *ft^EBR1/2^::FLAG (1-5)* and *(4-3)*. Genotypes were validated by sequencing.

*dlish^B1601^* was obtained from Seth Blair (University of Wisconsin-Madison). Transgenic RNAi *fbxl7:* *HMJ22830* was obtained from Bloomington (BL60461).

All crosses were raised at 25°C. Mitotic clones were induced using *hsFLP* by incubating the larvae at 37°C for 1 h, 48 h after egg laying (AEL) or for 1 h at both 48 and 72 h AEL. Specific genotypes described below:

FIG 1A-B”’, suppFig 1C-D’, suppFig 5A-B’: *hsFLP ;; FRT82B, ubi-mRFP.nls / FRT82B, crb^11A22^*

FIG 1D-D’: *tub-Gal4, hsFLP, UAS-nls.GFP::myc ;; FRT82B, tub-Gal80 / FRT82B, crb^11A22^*

FIG 1E-E’: *tub-Gal4, hsFLP, UAS-nls.GFP::myc ; UAS-Ft::HA / + ; FRT82B, tub-Gal80 / FRT82B, crb^11A22^*

FIG 1G-H”’: *hsFLP; ubi-GFP, FRT40A / ft^5^-5, FRT40A ; FRT82B, ubi-mRFP.nls / FRT82B, crb^11A22^*

suppFIG 1A-A’: *hsFLP; ex::GFP / + ; FRT82B, ubi-mRFP.nls / FRT82B, crb^11A22^*

suppFIG 1B-B’: *hsFLP; ex::GFP / + ; FRT82B, ubi-mRFP.nls / FRT82B, crb^82^-04*

suppFIG 1E-E’: *hsFLP; ubi-mRFP.nls, FRT40A / ex^e1^, FRT40A*

suppFIG 1F-F’, J-J’: *hsFLP; ubi-mRFP.nls, FRT40A / ft^5^-5, FRT40A*

suppFIG 1G-G”’: *; ex::GFP/ex::GFP*

suppFIG 1H-I”’: w^1118^

FIG 2D **– Genetic Control**, suppFIG 2B-B”’: ; ; *ubi-Ex^1^-468::GFP / ubi-Ex^1^-468::GFP*

FIG 2D, suppFIG 2C − **Ex::Ft Interaction:** *; ft::FLAG (2) / ft::FLAG (2) ; ubi-Ex^1-468^::GFP / ubi-Ex^1^-468::GFP*

FIG 4C: ; *ft::FLAG (2) / ft^fd^*

FIG 4D: ; *ft^EBR1^::FLAG (1-1) / ft^f^*

FIG 4E: ; *ft^ΔE^::FLAG (5-2) / ft^fd^*

FIG 4F: ; *ft^EBR1/2^::FLAG (4-3) / ft^fd^*

FIG 4G: ; *ft^EBR1^::FLAG (1-1) / ft^fd^,yki^B5^*

suppFIG 4HJ: ; *ft^ΔE^::FLAG (5-2) / ft^fd^,yki^B5^*

suppFIG 4I: ; *ft^EBR1/2^::FLAG (4-3) / ft^fd^,yki^B5^*

suppFIG 4B-B’, FIG 5A-A’, D-D’, G-G’: *hsFLP; ubi-mRFP.nls, FRT40A / ft^EBR1^::FLAG (1-1), FRT40A*

suppFIG 4C-C’, FIG 5B-B’, E-E’, H-H’: *hsFLP; ubi-mRFP.nls, FRT40A / ft^ΔE^::FLAG (5-2), FRT40A*

suppFIG 4D-D’, FIG 5C-C’,F-F’, I-I’: *hsFLP; ubi-mRFP.nls, FRT40A / ft^EBR1/2^::FLAG (4-3), FRT40A*

suppFIG 4A **(lane1)**, E: ; *ft::FLAG (2) / ft::FLAG (5)*

suppFIG 4A **(lane2)**, F: ; *ft^EBR1^::FLAG (1-1) / ft^EBR1^::FLAG (3-2)*

suppFIG 4A **(lane3)**, G: ; *ft^ΔE^::FLAG (1-2) / ft^ΔE^::FLAG (5-2)*

suppFIG 4A **(lane4)**, H-I: ; *ft^EBR1/2^::FLAG (1-5) / ft^EBR1/2^::FLAG (4-3)*

FIG 5J-K’, suppFIG 5E-I’: *hsFLP; ubi-mRFP.nls, FRT40A / ds^38k^, FRT40A*

suppFIG 5C-C’: ; *UAS-Dicer2 / UAS-fbxl7^IR^ (BL60461); enGal4, UAS-GFP / +*

suppFIG 5D-D’: ; *UAS-fbxl7^IR^ (BL60461) / + ; hh-Gal4, ubi-Ex^1^-468::GFP / +*

### Immunostaining

Imaginal discs were dissected, fixed using 4% formaldehyde (Thermo Fisher Scientific) for 20 minutes and stained as per standard protocols. Primary antibodies were incubated overnight at 4°C and secondary antibodies were incubated for 1-2 h at room temperature. Tissue was incubated with Hoechst 33342 (Thermo Fisher Scientific) for 5 minutes and mounted in ProLong Diamond Antifade (Thermo Fisher Scientific). Imaging was performed on a Nikon Ti2 confocal laser scanning microscope. XY confocal micrographs of third instar wing imaginal discs represent max-intensity projections of apical z-stacks merged with single sections at the nuclei. Primary antibodies: rat anti-Ci155 (2A1; DSHB), mouse anti-Crb 1:50 (Cq4; DSHB) − post-fixed tissue serially dehydrated with 30%, 50% and 70% methanol, rat anti-Crb 1:200 (kindly provided by F. Pichaud), rabbit anti-Ds 1:200 (DZ41169; Boster Biological Technology), rat anti-Dachs 1:1000, rabbit anti-Dlish 1:100 − preabsorbed with fixed *dlish^B1601^* homozygous mutant larvae (both kindly provided by S. Blair), guinea pig anti-Ex 1:1000 − preabsorbed with fixed *ex^e1^* homozygous mutant larvae (kindly provided by R. Fehon), rat anti-Ft 1:500 (Sopko et al., 2009) and rat anti-HA 1:250 (3F10; Roche Applied Science). Secondary antibodies used 1:1000: goat anti-guinea pig Alexa Fluor 488, goat anti-guinea pig Alexa Fluor 647, donkey anti-mouse Alexa Fluro 488, donkey anti-rabbit Alexa Fluro 488, donkey anti-rat Alexa Fluor 594 and donkey anti-rat Alexa Fluor 647 (all Thermo Fisher Scientific).

### Cell Culture, Transfection and Expression Construct Generation

*Drosophila* S2R+ cells were cultured at 25°C in Schneider’s S2 media (Thermo Fisher Scientific) supplemented with 10% foetal bovine serum (FBS, Sigma Aldrich), 100U/mL Penicillin and 100 µg/mL Streptomycin (Thermo Fisher Scientific). DNA was transfected using the Effectene reagent (Qiagen). HEK293 cells were cultured at 37°C and 5% CO_2_ in DMEM (Thermo Fisher Scientific) supplemented with 10% FBS (Sigma Aldrich), 1% GlutaMAX (Thermo Fisher Scientific), 100U/mL Penicillin and 100 µg/mL Streptomycin (Thermo Fisher Scientific). DNA was transfected using the Lipofectamine 2000 (Thermo Fisher Scientific) or Lipofectamine 3000 reagents (Thermo Fisher Scientific).

Cloning into cell expression vectors was performed us using standard PCR/restriction enzyme-based cloning, Gateway technology (Thermo Fisher Scientific), Q5 Site-Directed Mutagenesis (New England BioLabs) or the pCDNA3.1/V5-His TOPO TA Expression Kit (Thermo Fisher Scientific) and confirmed by sequencing.

#### Expression constructs

pAWF Ex^FL^ and pAWF Ex^FERM^ (FERM domain defined as amino acids 1-400) were previously described (Badouel et al., 2009). pAc Ft^ΔECD^ was previously described (Matakatsu & Blair, 2006) and subcloned to pCMV5 Ft^ΔECD^::FLAG (Sopko et al., 2009) from which all further pCMV5 Ft^ΔECD^::FLAG constructs were subcloned. All vectors were verified by sequencing.

#### Constructs made for this study

##### Lysate Preparation and Immunoprecipitation

S2R+ lysates were generated using lysis buffer: 50 mM Tris pH 7.5, 150 mM NaCl, 1% Triton X-100, 10% glycerol and 1 mM EDTA supplemented with HALT protease and phosphatase inhibitor cocktail (Thermo Fisher Scientific). HEK293 lysates were generated using lysis buffer: 50 mM Hepes, 100 mM KCl, 2mM EDTA, 0.1% NP40 (IGEPAL CA-630) and 10% glycerol supplemented with HALT protease and phosphatase inhibitor cocktail (Thermo Fisher Scientific). *Drosophila* L3 larval wing disc lysates were generated using 2x Laemmli buffer (Bio-Rad). FLAG-immunopurification was performed by incubating cell extract with anti-FLAG M2 affinity agarose gel (Sigma Aldrich) for ≥2 h at 4°C under agitation. FLAG beads were subsequently washed with lysis buffer and eluted by incubation with 2x SDS sample buffer for 4 min at 95°C or by elution using 150 ng/µl FLAG peptide (Sigma Aldrich) for 30-60 min.

##### Immunoblotting

Lysates were analysed by standard chemiluminescent immunoblotting techniques. Primary antibodies: mouse anti-actin 1:2000 (C-4; Sigma Aldrich), mouse anti-Biotin 1:400 (33; Santa Cruz Biotechnology), rat anti-Ft 1:500 (Sopko et al., 2009), mouse anti-FLAG 1:5000 (M2; Sigma Aldrich), mouse anti-GST 1:500 (B-14; Santa Cruz Biotechnology), rabbit anti-HA 1:1000 (C29F4; Cell Signaling Technology), rat anti-HA 1:2000 (3F10; Roche Applied Science) and mouse anti-V5 1:5000 (R960-25; Thermo Fisher Scientific).

##### Proximity Ligation Assay

PLA was performed using the Duolink kit (Sigma Aldrich) as per manufacturer’s protocol. Wing discs were incubated with PLA probes for 2 h at 37°C, ligase for 1 h at 37°C and polymerase for 2 h at 37°C. Samples were stained with Hoechst 33342 (Thermo Fisher Scientific) and mounted in ProLong Diamond Antifade (Thermo Fisher Scientific). Primary antibodies: mouse anti-FLAG 1:250 (M2; Sigma Aldrich), rabbit anti-GFP 1:1000 (ab290 Abcam).

##### GST expression and purification

GST-proteins were transformed into Rosetta (DE3) cells (Sigma Aldrich) and induced by incubation with 0.1 mM IPTG at 18°C. Bacteria were lysed using B-PER Complete (Thermo Fisher Scientific) supplemented with HALT protease and phosphatase inhibitor cocktail (Thermo Fisher Scientific) and GST-tagged protein was isolated using Glutathione Sepharose (Sigma Aldrich) and eluted using 10 mM reduced glutathione, 50 mM Tris-HCl pH 8. Buffer exchange into PBS was performed using Zeba Spin Desalting Columns (Thermo Fisher Scientific).

##### Binding assay between GST, Biotin-tagged or FLAG-tagged and *in vitro* translated protein

*In vitro* Dlish, GFP, Ex^1^-468, Ex^NT^, Ex^CT^ and Ds^ICD^ were generated using TnT T7 Quick Coupled Transcription/Translation System (Promega) as per the manufacturer’s protocol. N-terminally Biotin-tagged Ft peptide was generated by GenScript. 100 pmol purified GST-protein, 10 ug Biotin-peptide or 10 uL FLAG-tagged TnT T7 product was incubated with 10 µL TnT T7 product in a total of 300 µL binding buffer: 0.02% NP40 (IGEPAL CA-630), 10% glycerol, PBS supplemented with 0.5 mM DTT 0.1 mM PMSF and HALT protease and phosphatase inhibitor cocktail (Thermo Fisher Scientific) for 2 h at 4°C. GST-protein binding reactions were then incubated with Glutathione Sepharose (Sigma Aldrich) and Biotin-peptide binding reactions were incubated with Pierce Streptavidin Agarose (Thermo Fisher Scientific) for 2 h at 4°C before purification by centrifugation. Beads were washed with PBS and were eluted by incubation with 2x Laemmli, 5% 2-Mercaptoethanol at 95°C for 8 min. FLAG-binding reactions were incubated with anti-FLAG M2 affinity agarose gel (Sigma Aldrich) overnight at 4°C before purification by centrifugation. Beads were washed with PBS and eluted by incubation with 150 ng/µl FLAG peptide (Sigma Aldrich) for 30-60 min. 2x SDS sample buffer was added to supernatant and incubated for 5 min at 95°C. Binding was analysed by immunoblotting.

##### Pupal lethality analysis

The percentage of pupal lethality was calculated by counting the total number of pupal cases and the number of non-eclosed pupal cases from the same vials and presented as a ratio. Statistical analysis by one-way ANOVA with the Dunnett’s post-hoc test was performed in GraphPad Prism.

##### Adult wing analysis

Adult *Drosophila* were collected in 70% ethanol. Wings were removed in isopropanol, mounted in Euparal (Anglian Lepidopterist Suppliers) and baked at 65°C for ≥5 h. Imaging was performed using a Leica M165 FC stereo microscope and wing parameters were quantified using ImageJ. The L3 vein was used to calculate wing length, and the distance between the distal ends of the L2 and L5 veins was used to calculate wing width, which were presented as a ratio to measure shape. Statistical analysis by one-way ANOVA with the Tukey’s or Dunnett’s post-hoc test was performed in GraphPad Prism.

##### Immunofluorescence Quantification and Processing

For quantification of apical fluorescence inside vs outside a clone, regions of interest were manually defined using the fluorescent clonal marker. Apical or basal mean pixel intensity was measured using NIS-Elements (Nikon) or Image J. Data points represent the averaged signals from at least two transverse sections per wing disc normalised to the average signal from the wild-type tissue. Statistical analysis by unpaired t-test or one-way ANOVA with the Tukey’s post-hoc test was performed in GraphPad Prism. Where indicated, images were denoised using NIS-Elements (Nikon).

## Supplementary Material

Supplementary Material

## Figures and Tables

**Table:** 

Construct name	Amino Acid (aa) residues
pAFW Ds^ICD^	aa:3115-3556*
pCDNA3.1 FLAG::Ds^ICD^	aa:3115-3556*
pGEX4T1 Ft^ICD^	aa:4717-5147*
**pCDNA3.1 C-terminal V5/His constructs:**	
GFP	n/a
Dlish	n/a
Ex^NT^	aa:1-653
Ex^CT^	aa:654-1427*
Ex^1-468^	aa:1-468
**pCMV5 Ft-C-terminal FLAG-tagged constructs:**	
cA244	aa:AECD-4903
cA255	aa:AECD-4892
myr-c124	aa:5024-5147*
myr-c124;AEBR2	aa:5024-5147*;A5084-5123
cA492	aa:AECD-4655
AEBR1-EBR2	aa:AECD-4892;5124-5417*
CA505-125	aa:AECD-4642;5024-5147*
cA492-256;A153	aa:AECD-4655;4893-4994
cA492-256;AC;A153	aa:AECD-4655;4893-4920;4951-4994
cA492-256;AD-CT	aa:AECD-4655;4893-4973
cA492-256;AC,AD-CT	aa:AECD-4655;4893-4920;4951-4973
