# Supplementary material for: Expanded directly binds conserved regions of Fat to restrain growth via the Hippo pathway
Source: J Cell Biol. Author manuscript; Available in PMC 2023 May 1. (PMC10120405; doi:10.1083/jcb.202204059)
Supplement: Supplementary Material [file EMS174323-supplement-Supplementary_Material.docx]

## Supplementary Figure Legends

Supplementary Figure 1 – Ft and Crb regulate Ex independently

**(A-B’)** A portion of Ex::GFP remains at the apical membrane despite loss of Crb. XY confocal micrographs of Ex::GFP third instar wing imaginal discs containing *crb^11A22^* (A-A’) or *crb^82-04^* (B-B’) mutant clones (marked by absence of RFP shown in red), showing direct GFP fluorescence representing Ex protein (shown in grey).

**(C-D’)** Validation of *crb^11A22^* as a protein null. XY confocal micrographs of third instar wing imaginal discs containing *crb^11A22^* mutant clones (marked by absence of RFP shown in red) with immunostaining to the extracellular domain (ECD) of Crb (C-C’ – shown in grey) or the intracellular domain (ICD) of Crb (D-D’ – shown in grey).

**(E-E’)** Validation of Ex immunostaining. XY confocal micrographs of third instar wing imaginal discs containing *ex^e1^* mutant clones (marked by absence of RFP shown in red) with Ex staining (shown in grey).

**(F-F’)** Loss of Ft causes loss of apical Ex. XY confocal micrographs of third instar wing imaginal discs containing *ft^5-5^* mutant clones (marked by absence of RFP shown in red) with Ex staining (shown in grey). *ft^5-5^* is a remake of *ft^fd^* and is a null allele.

**(G-G’’’)** Colocalisation of Ex, Ft and Crb at apical junctions. Transverse confocal micrograph of third instar wing imaginal discs with direct fluorescence of Ex::GFP (green in G and G’’’), stained with Ft (mauve in G’ and G’’’), Crb (red in G’’ and G’’’) and Hoechst (cyan in merge) to mark nuclei.

All images are orientated dorsal up. Clonal boundaries are marked by yellow dotted lines. Scale bars are 10 µm.

**(H-I’’)** High resolution localisation patterns of Ex and Ft with even distribution (H-H’’) or punctate (I-I’’). High resolution XY confocal micrographs of third instar wing imaginal discs stained for Ft (green in H’’ and I’’) and Ex (mauve in H’’ and I’’). The yellow arrows in (I-I’’) highlight punctate colocalisation. Images were denoised, scale bar is 2 µm.

**(J-J’)** Loss of Ft does not affect Crb. XY confocal micrographs of third instar wing imaginal discs containing *ft^5-5^* mutant clones (marked by absence of RFP shown in red) with Crb staining (shown in grey). *ft^5-5^* is a remake of *ft^fd^* and is a null allele. Images are oriented dorsal up. Scale bar is 10 µm.

**(K)** Quantification of the ratio between basal Ex inside versus outside the MARCM clone normalised to the wildtype tissue. Data points represent an average of a single disc with the mean and standard deviation indicated. No significance calculated using an unpaired T-test.

Supplementary Figure 2 – Ft and Ex directly bind at the apical membrane

**(A)** Ft^ICD^ directly binds Ex^1-468^. *In vitro* transcribed and translated Ex^1-468^ was incubated with bacterially expressed and purified GST alone or GST::Ft^ICD^ and subjected to GST-purification.

**(B-B’’)** High resolution colocalisation of Ex^1-468^ and Ft. XY confocal micrograph of third instar wing imaginal discs stained with Ft (mauve B’’) with direct fluorescence of Ex^1-468^::GFP (green in B’’).

**(C)** Ft and Ex interact at apical membrane *in vivo*. XY confocal micrographs of third instar imaginal discs expressing *ubi-Ex^1-468^::GFP* subjected to anti-FLAG and anti-GFP PLA. Ex::Ft interaction condition expresses *ft::FLAG* at the endogenous locus and *ubi-Ex^1-468^::GFP*. Ex^1-468^::GFP is observed by direct fluorescence of GFP (grey or green in merge), PLA signal (grey or red in merge) mark interaction loci, which overlap with Ex signal. Images were denoised, scale bar is 2 µm.

**(D)** Ft^ICD^ does not directly binds Dlish. *In vitro* transcribed and translated Ex^1-468^ (as a positive control) and Dlish were incubated with bacterially expressed and purified GST alone or GST::Ft^ICD^ and subjected to GST-purification.

The expression and presence of proteins was analysed by immunoblotting with the indicated antibodies.

Supplementary Figure 3 – Identification of Expanded Binding Regions within the Ft-ICD

**(A)** Identification of Expanded Binding Region (EBR) 2 in the Ft-ICD. HEK293 cell expression and IP of indicated FLAG-tagged Ft^ΔECD^ constructs in the presence of Ex^FERM^.

**(B)** Ft conserved-C and -D regions affect interaction with Ex. HEK293 cell expression and IP of indicated FLAG-tagged Ft^ΔECD^ constructs in the presence of Ex^FERM^.

The expression and presence of proteins was analysed by immunoblotting with the indicated antibodies. Ft presents as multiple bands due to proteolytic processing (Feng & Irvine, 2009; Sopko et al., 2009).

**(C)** Graphical scheme highlighting the Ft constructs used in figure supplement 3. In addition, the transmembrane domain (TM), EBR1, EBR2 and established conserved and function domains of the Ft-ICD are depicted. In binding column: ‘++’ denotes constructs that interact strongly to Ex^FERM^, ‘+’ denotes weak interaction with Ex^FERM^ and ‘-’ denotes no interaction with Ex^FERM^.

Supplementary Figure 4 – EBRs are required *in vivo* for regulation of tissue growth

**(A)** EBR deletion does not affect Ft levels. Immunoblot of trans-heterozygous EBR allele L3 wing discs. Compared to cntrl (ft::FLAG), there is no change in Ft levels in the EBR alleles. Proteins were analysed by immunoblotting with the indicated antibodies. Actin was used as a loading control. Ft presents as multiple bands due to proteolytic processing (Feng & Irvine, 2009; Sopko et al., 2009).

**(B-D’)** EBR deletion does not affect Ft levels or localisation. XY confocal micrographs third instar wing imaginal discs containing of *ft^EBR1^* (B-B’), *ft^ΔE^* (C-C’) or *ft^EBR1/2^* (D-D’) mutant clones (marked by absence of RFP shown in red) with Ft staining (shown in grey). XY images are orientated as dorsal up. Clonal boundaries are marked by yellow dotted lines. Scale bars are 10 µm.

**(E-I)** Effect of trans-heterozygous EBR deletion on adult wing phenotypes. Phenotype of control (cntrl) - ft::FLAG (E), ft^EBR1^ (F), ft^ΔE^ (G), bulk ft^EBR1/2^ (class 1) (H). or ft^EBR1/2^ (class 2) (I) wings.

**(J)** Quantification of pupal lethality in trans-heterozygous EBR animals. Compared to cntrl (ft::FLAG) wings, ft^ΔE^ and ft^EBR1/2^ cause a significant increase in pupal lethality. Data points indicate one vial of approximately 30 pupae, with mean and standard deviation represented. ***P<0.005 using one-way ANOVA with a Dunnett’s post-hoc test compared to the ft::FLAG control. ns denotes non-significant.

**(K)** Quantification of adult wing size in trans-heterozygous EBR flies. Data are normalised against the mean of the cntrl (ft::FLAG). ft^EBR1^ causes significant overgrowth. ft^EBR1/2^ ‘class 2’ flies are isolated from the bulk ft^EBR1/2^ to indicate the significant overgrowth. Data points indicate an individual wing with mean and standard deviation represented. ****P<0.0001 using one-way ANOVA with a Dunnet’s post-hoc test compared to cntrl. ns denotes non-significant.

**(L)** Quantification of adult wing roundness in trans-heterozygous EBR flies. Shape was determined by the ratio of wing length verses width. Data are normalised against the mean of the cntrl (ft::FLAG). ft^EBR1^ and ft^ΔE^ are significantly rounder than cntrl. ft^EBR1/2^ ‘class 2’ flies are isolated from the bulk ft^EBR1/2^ to indicate the significant increase in roundness. Data points indicate an individual wing with mean and standard deviation represented. ***P=0.0004 and ****P<0.0001 using one-way ANOVA with a Dunnet’s post-hoc test compared to cntrl. ns denotes non-significant.

Supplementary Figure 5

**(A-B’)** Loss of Crb has no effect on Dachs and Dlish. XY confocal micrographs of third instar wing imaginal discs containing *crb^11A22^* mutant clones (marked by absence of GFP shown in green) with Dachs staining (A-A’ - shown in grey) and Dlish staining (B-B’ - shown in grey).

**(C-C’)** RNAi mediated knockdown of Fbxl7 has no effect on Ex. XY confocal micrographs of third instar wing imaginal discs where *en-Gal4* was used to drive expression of *UAS-Dicer2*,*UAS-GFP*,*UAS-fbxl7^IR^* with Ex staining (shown in grey). GFP marks the Gal4 positive posterior compartment (shown in green).

**(D-D’)** RNAi mediated knockdown of Fbxl7 has no effect on ubi-Ex^1-468^::GFP. XY confocal micrographs of third instar wing imaginal discs where *hh-Gal4* was used to drive expression of *UAS-fbxl7^IR^* with direct GFP fluorescence representing ubi-Ex^1-468^::GFP (shown in grey). Cubitus Interruptus (Ci) staining marks the Gal4 negative anterior compartment (shown in red).

**(E-E’)** Validation of *ds^38K^* as a protein null. XY confocal micrographs of third instar wing imaginal discs containing *ds^38k^* mutant clones (marked by absence of RFP shown in red) with Ds staining (shown in grey).

**(F-G’)** Loss of *ds* dramatically increases apical Dachs and Dlish. XY confocal micrographs third instar wing imaginal discs containing *ds^38k^* mutant clones (marked by absence of RFP shown in red) with Dachs staining (D-D’ - shown in grey) and Dlish staining (E-E’ - shown in grey).

**(H-H’)** Loss of *ds* has no effect on Crb. XY confocal micrographs third instar wing imaginal discs containing *ds^38k^* mutant clones (marked by absence of RFP shown in red) with Crb staining (shown in grey).

**(I-I’)** Loss of *ds* has a minimal effect on Ft in the pouch. XY confocal micrographs third instar wing imaginal discs containing *ds^38k^* mutant clones (marked by absence of RFP shown in red) with Ft staining (shown in grey).

All XY images are orientated as dorsal up. Clonal boundaries are marked by yellow dotted lines. Scale bars are 10 µm.

**(J)** Ds^ICD^ does not bind to Ex^NT^ or Ex^CT^. *In vitro* transcribed and translated Ex^NT^ or Ex^CT^ were incubated alone or with *in vitro* transcribed and translated Ds^ICD^ and subjected to FLAG-immunoprecipitation.

**(K)** Ds^ICD^ does not bind to Dlish. *In vitro* transcribed and translated Dlish was incubated alone or with *in vitro* transcribed and translated Ds^ICD^ and subjected to FLAG-immunoprecipitation.

The expression and presence of proteins was analysed by immunoblotting with the indicated antibodies.
